# From Dr. G. W.

**Published:** 1875-08

**Authors:** 


*From Dr. G. W.*

I notice with much gratification an editorial from the Senior Editor in reply to J. C. C., in reference to laws in relation to the practice of medicine, the vending of drugs, and the powers conferred upon the State Medical Board.

If the version of Dr. Powell (who is a member of the Board,) be true, then the law is a good one, calculated to give ample protection to the citizens and to elevate the dignity of the profession. Now, I and all physicians know that the evils the law is designed to correct are in full operation. I therefore see very clearly that the fault is neither with the Board or the law, but consists in the failure of physicians all over the State to perform their duty, or, rather, see that the grand juries of every county take the matter in hand and compel all who have licenses and practice medicine and vend medicines, to have them recorded, and indite all who have not procured them, in violation of the law.

I regret that the law requiring physicians who have diplomas to exhibit them to the Board, and then obtain licenses, was repealed. This feature of the law was repealed because it seemed to make the Board the controllers of the profession; to give them the power to say who should and who should not practice medicine or vend drugs in the State. I am satisfied that this was a misconception and an error. I believe the object was for the protection of all physicians, who would, in aiding the law, gladly exhibit their diplomas, in order that the quacks and others who had no such showing might be legally debarred from engaging in the practice, and show to the grand juries who were violating the law, who were impostors, and who should be liable to prosecution under the law. For this reason no one was particularly charged with the prosecution of violators of the law, because the recording of licenses, which reveals the fact of violation, and gives the grand juries no trouble in ascertaining the facts.

I am satisfied that the view Dr. Powell takes of the law is correct, but would respectfully ask the Doctor to give further light upon the following language. I quote from the editorial:

"We hold, furthermore, that the spirit of the law makes it obligatory upon every practitioner to practice strictly in accordance with the medical system under which he holds his diploma, whenever and wherever he claims the right to practice under the shelter of said diploma, and we maintain that he becomes a violator of the law whenever he engages in practice which his Alma Mater would disclaim as quackery and foreign to its teachings and subversive of its doctrines. This mal-practice and quackery exists in the State, doubtless, but, as before said, the Examining Board have no prosecuting jurisdiction in such cases, as an official body. It, therefore rests with the people themselves, and their grand juries, who alone have power to rid the community of these nuisances, and prevent the violation of the law for the protection of the public health."

This is the road, if I understand it, to the very worst form of quackery, and it makes the law invaluable in its protection to the profession and the people of the State. It not only does this, but it makes a test of the faithfulness of Medical Colleges in protecting their graduates, and in having to keep inviolate the ethics of the profession.

Will the Doctor give me his views on the above, and oblige?

---

We publish the above letter, written by one of the ablest physicians of Georgia, in the hope that it may throw additional light upon the important subject of the law governing the practice of medicine in this State.

Every good physician is deeply interested in the matter, and we earnestly trust that the study which one correspondent has evidently devoted to the subject, will arouse a similar spirit of investigation in all who desire to promote the best interest of the profession in our State.

The law is plain and easily comprehended, and it is the solemn duty of every physician, as well as every good citizen, to see to it that its privileges are unobstructedly enjoyed, and that its provisions for guarding the health of the people, and the honor of the profession, are promptly and sacredly enforced. As soon as the law is enforced it will not be necessary for the officers of the law to intervene for the purpose of removing the grave evils which exist, because the very fact that punishment is ever imminent upon violations, the mere dread of the penalty will prevent the perpetration of illegal and pernicious actions on the part of imposters and quacks.

The Board of Medical Examiners has appointed a committee whose duty it is to call the attention of the various Judges and Attorney Generals throughout the State to the provisions of this law, requesting them to particularly charge the grand juries upon this subject.

This action is as far as the Board can legally go, and will be recognized by all as a full discharge of this duty in the premises.

If our judges and judicial officers generally will do their full duty in this particular, and if physicians, in each county, upon whom the welfare of their community and the high standard of their profession rests, will also do their duty, then the great and beneficial object of this most important law will be easily accomplished, and the people of Georgia will reap all of its benefits.

Since the publication in the June number of the RECORD, of our reply to Dr. J. C. C., we have submitted the interpretation of the law, as therein given, to one of our most learned lawyers, who says our views are fully sustained by the meaning and intent of the law.

In regard to the paragraph, of which our correspondent requests further light, we would say that we hold that a physician who practices in a manner not authorized by his diploma, or in a manner not endorsed by the college from which he received this diploma, and which he holds as a legal protection for his practice, he violates not only his diploma, but subjects himself to the penalties of our State law, just as if he were practicing without any diploma whatever. Suppose Dr. A., holding a diploma from a college of the first order in this country, comes to Georgia, and, from mercenary motives, he engages in practice which is violative of the intentions and principles of his college diploma, and destructive of the ethics of his school by unprofessional tricks of charlatanism. Upon this he is arraigned before the grand jury as a violator of the State law governing the practice of physicians, he presents his diploma as his authority, then it becomes the duty of the officers of the law to demand that he prove that he has, in no instance, violated the integrity of his diploma, and if he admits that he has, he must give proof that his college endorses all of his departures from the strict letter and spirit of the diploma with which it invested him. This would be the only way for such a dependent to clear himself from the penalties prescribed, and to make his diploma, under such circumstances, valid.

Any college, however, which would knowingly endorse one of of its graduates guilty of quackery, or refuse to revoke his diploma, might subject itself to being tabooed by other colleges of good standing—that subject, of course, would be a matter of investigation and debate for the Medical Association of the country. As a member of the Board of Medical Examiners of Georgia, we do not propose to discuss the merits of this branch of the case. Our object is to show that neither of the Examining Boards in Georgia can license a person to practice a system of medicine different from the one it represents, and that a diploma gives no protection in the practice of a system not recognized and endorsed by the college from which that diploma was obtained, no more than a druggist can sell liquors by the retail under his license as a druggist. Our object is also to show that the honor of our profession as well as the safeguard against quackery lies in the hands of every true physician in the State, as well as in some degree, in that of every good citizen.

Let those who are impressed with this view of the matter go to work in earnest, and remove the serious evils now so generally complained of, and for which, heretofore, the Board has, in a great degree, been held responsible. P.

*Medical Colleges.*

